# Cement leakage in percutaneous vertebroplasty for spinal metastases: a retrospective study of risk factors and clinical outcomes

**DOI:** 10.1186/s12957-022-02583-5

**Published:** 2022-04-07

**Authors:** Lin Wang, Chao Zhang, Hao Liang, Tianji Huang, Weiyang Zhong, Zenghui Zhao, Xiaoji Luo

**Affiliations:** 1 Department of Orthopedic Surgery, The First Affiliated Hospital of Chongqing Medical University, Chongqing, 400016 People’s Republic of China; 2 Orthopedic Laboratory of Chongqing Medical University, Chongqing, 400016 People’s Republic of China

**Keywords:** Percutaneous vertebroplasty, Cement leakage, Spine metastases, Risk factors

## Abstract

**Objective:**

The objective of this research was to investigate the risk factors of cement leakage in patients with metastatic spine tumors following percutaneous vertebroplasty (PVP).

**Methods:**

Sixty-four patients with 113 vertebrae were retrospectively reviewed. Various clinical indexes, including age, sex, body mass index (BMI), smoking history, drinking history, chemotherapy history, radiotherapy history, primary cancer, location, other metastases, collapse, posterior wall defects, the laterality of injection, and the injected cement volume were analyzed as potential risk factors. Multivariate analyses were conducted to identify the independent risk factors.

**Results:**

The cement leakage was found 64 in 113 treated vertebrae (56.63%), in which the incidence of each type was shown as below: spinal canal leakage 18 (15.93%), intravascular leakage around the vertebrae 11 (9.73%), and intradiscal and paravertebral leakage 35 (30.97%). Tomita classification (*P* = 0.019) and posterior wall destruction (*P* = 0.001) were considered strong risk factors for predicting cement leakage in general. The multivariate logistic analysis showed that defects of the posterior wall (*P* = 0.001) and injected volume (*P* = 0.038) were independently related to the presence of spinal canal leakage. The postoperative visual analog scale (VAS) and activities of daily living (ADL) scores showed significant differences compared with the pre-operative parameters (*P* < 0.05). No significant differences were found in every follow-up time between the leakage group and the non-leakage group for pain management and improvement of activities in daily life.

**Conclusion:**

In our study, Tomita classification and the destruction of the posterior wall were independent risk factors for leakage in general. The defects of the posterior wall and injected volume were independently related to the presence of spinal canal leakage. The PVP procedure can be an effective way to manage the pain.

## Introduction

The spine is considered one of the most common tumor metastatic sites, and the incidence rates increase year by year due to the development of medical services capabilities. According to statistics, the vast majority of metastases happened in the thoracic spine (70%), while the lumbar spine (20%) and cervical spine (10%) make up the rest [1]. Depending on the exact size, location, and extent, patients may suffer from severe pains, deficits in the abilities of daily life, and neurological symptoms, in which pain is the predominant one [2]. Traditionally, analgesics, radiotherapy, and surgical procedures can be applied to relieve the pain. Among them, surgical procedures were considered the first choice, especially when spinal instability led to axial pain or spinal cord compression occurred. Compared with open surgery, minimally invasive surgeries like percutaneous vertebroplasty (PVP) had innate advantages of less trauma and time for those patients with short life expectancy [3–5].

PVP is a minimally invasive, image-guided therapy that is widely accepted and adopted for palliative treatment. The polymethyl methacrylate (PMMA) bone cement is injected at sites of vertebral lesions by a percutaneous puncture to restore vertebral height and stability. It had been used to treat osteoporotic vertebral collapse [6], benign lesions (vertebral angiomas [7], monostotic fibrous dysplasia [8], etc.), and malignant tumors [9]. According to previous research, PVP was proven to be an effective and safe way to relieve pain [2, 5] and stabilize the vertebrae when applied alone or combined with some novel strategies [10–12]. Moreover, patients treated with PVP needed less recovery due to the small trauma. Thereby, it was considered the ideal choice for patients whose general condition is relatively poor, with decreased pain, less blood loss, and shorter hospital stays [5].

The major complication after PVP is cement leakage, as many previous studies had found [6, 9, 13–15]. Although it may not result in symptoms in an overwhelming number of cases. Based on the existing studies about osteoporotic vertebra compression fractures (OVCF) following PVP, the incidence of cement leakage ranged from the lowest 5% to 80% for the highest. However, the incident rate of that in malignant tumors varies from 50% to nearly 100% due to the potential posterior wall deficits and rich blood supply [6, 9, 14, 16]. To our best knowledge, there is still little research to explore the risk factors for cement leakage in spine metastases.

Therefore, in this study, we aim to evaluate potential risk factors for cement leakages in patients with spinal metastases after PVP, evaluate the therapeutic capacity of PVP, and analyze the relationship between clinical parameters and the occurrence or not of leakage.

## Material and methods

### Selection of patients

This study was approved by the ethics committee of The First Affiliated Hospital of Chongqing Medical University. And the informed consent was waived for the retrospective study. We retrospectively reviewed patients with metastatic spinal tumors treated after PVP at our department between Jan. 2015 to Dec. 2020. The inclusion criteria are shown as follows: (1) spine metastases confirmed by histopathological diagnosis using biopsy; (2) severe and intractable pains which can be attributed to the lesion; (3) treated with PVP; (4) osteolytic or mixed lesions. Exclusion criteria were (1) primary spinal tumor, (2) spinal cord and/or nerve root compression, (3) pathological vertebral due to tuberculosis, (4) vertebral fractures due to another cause such as osteoporosis, and (5) the previous history of receiving PVP.

The major surgical indication for all patients was the unbearable pain due to the osteolytic destruction at the level of pathological vertebral sites. Moreover, the symptoms could not be relieved by any formal conservation treatments.

## Methods

### Surgical procedure

In our study, all the surgeries were conducted by the senior spinal surgeons in our department. The procedure was performed in a prone position under local anesthesia. The initial image was acquired with the help of C-arm to locate the targeted site. Under the guidance of C-arm X-ray (Ziehm Imaging Systems), the 11- or 13-gauge puncture needle was unilaterally or bilaterally inserted through the pedicle until it reached the posterior one-third of vertebrae, then advanced into the vertebral body. In this stage, the diseased vertebral tissue could be taken for biopsy through the walking tunnel. Finally, polymethylmethacrylate (PMMA) was slowly injected into the fractured vertebral body. The cement volume and complications were recorded accordingly.

### Data collection

#### Pre-operative patients’ demographics

The following data were obtained from the electronic medical record system: age, sex, body mass index (BMI), smoking history, alcohol-drinking history, primary cancer type, chemo/radiotherapy history, other metastases, number of pathological vertebrae, collapse, the destruction of the posterior wall, the injected volume of PMMA, instrumentation (single or bilateral), whether leakage or not. The primary tumor types were categorized into three subtypes: slow, moderate, and rapid groups, according to the Tomita classification. Widely accepted and adopted to determine the optimal treatment for patients with spine metastases [17], the Tomita classification distinguished the degree of malignancy and prognosis by the growth speed of primary cancer type [18]. The specific groups are as follows: (1) slow growth group (breast, thyroid, prostate, and so on.); (2) moderate growth group (kidney, uterus, and so on); (3) rapid growth group (lung, liver, and so on) [18].

#### Clinical evaluation

Postoperative parameters, including the visual analog scale (VAS) score [19] and activities of daily living (ADL) score [20] based on the Barthel Index, were recorded. The Barthel Index is a 10-item measurement of ADL. A higher Barthel index score indicates a superior capacity of daily living. The leakage of cement was assessed by the third party who had not participated in the surgeries. For all cases, the following data observed in three time periods which included preoperative, one month postoperatively, and the final follow-up (3 months) are shown below: whether leakage or not, VAS, and ADL.

#### Assessment of cement leakage

All patients underwent an X-ray and CT scan of the spine after the procedure. The leakage of cement was evaluated and recorded as three subtypes: (1) spinal canal leakage; (2) intravascular leakage around the vertebrae; (3) intradiscal and paravertebral leakage.

#### Analysis

The analysis was performed using the IBM SPSS Statistics 26.0. The D’Agostino-Pearson test was used to check the normality of distribution for measurement data which were shown as number (%), mean ± standard deviation. The intra-group comparison was conducted by independent sample *t*-test. One-way ANOVA was used to compare the difference between multi-groups. Non-normal distributed variables were presented as medians and interquartile range (IQR). The comparisons between groups were performed through the Mann–Whitney *U* test and the Kruskal–Wallis test. All count data were expressed as ratio and compared using a chi-square test or Fisher’s exact probability method. Differences with a *P*-value < 0.05 were considered significant.

## Results

This study reviewed the medical history, pathology, and imaging data of 64 patients (36 males and 28 females) with 113 pathological vertebrae involved. The median age was 65 years (range: 27–89). Among all the patients involved, 42 patients (65.63%) were diagnosed with rapidly growing cancer. 28 patients (46.88%) reported receiving standard chemoradiotherapy prior to this surgical procedure. Moreover, postoperative chemotherapy and radiotherapy were adapted by 45 patients (73.44%). Of all the involved vertebrae, the lumbar spine accounted for 66 (58.41%), accounting for the highest, thoracic spine accounted for the remaining 47 (41.59%). The collapse or pathological fracture occurred in 44 vertebrae. 89 of all the treated vertebrae presented with posterior wall defects. The patients’ demographics and clinical and radiographic features are shown in Table 1.

**Table 1:** The patients’ demographics and clinical and radiographic features

No of patients	64
No. of treated vertebrae	113
Gender	
Male	36
Female	28
Mean age (range, years)	65 (27–89)
BMI (range, kg/m^2^)	(16.16–30.47)
Smoking history	
Yes	28
No	36
Drinking history	
Yes	19
No	45
Previous chemo/radiotherapy	
Yes	28
No	36
Post-OP chemo/radiotherapy	
Yes	45
No	19
Primary cancer type	
Slow	14
Moderate	8
Rapid	42
Other metastases	
Yes	19
No	45
Number of metastatic vertebrae (range)	(1–27)
Treated vertebra level	
Thoracic	47
Lumbar	66
Collapse	
No	69
Yes	44
Posterior wall destruction	
Yes	89
No	24
Injected bone cement volume (range, ml)	4.84 ± 1.70 (1.2–9)

A total of 64 vertebrae was found suffering from bone cement leakage, in which the incidence of each type was shown as below: spinal canal leakage 18 (15.93%), intravascular leakage around the vertebrae 11 (9.73%), and intradiscal and paravertebral leakage 35 (30.97%). Table 2 demonstrates the univariate analysis of cement leakage in general, in which six factors were confirmed to be the risk factors. These factors included Tomita classification (*P* = 0.026), postoperative chemoradiotherapy (*P* = 0.002), the level of treated vertebrae (*P* = 0.03), posterior wall destruction (*P* < 0.001), injected laterality (*P* = 0.002) and injected volume (*P* = 0.007). Then, we conducted a multivariate logistic analysis to find out the factors that are independently related to cement leakage. Two factors were validated to be statistically significant: Tomita classification (odds ratio (OR) = 2.060, 95% confidence interval (CI) = 1.124-3.776, *P* = 0.019) and posterior wall destruction (OR = 19.706, 95% CI = 3.653-106.297, *P* = 0.001) (Table 3). Besides that, we also found that injected laterality (*P* = 0.086) and injected volume (*P* = 0.066) approached statistical significance.

**Table 2:** Univariate analysis for the occurrence of cement leakage in general

Features	No leakage = 49	Leakage = 64	*P*
Previous chemo/radiotherapy			0.414
No	23 (46.9%)	35 (54.7%)	
Yes	26 (53.1%)	29 (45.3%)	
Tomita classification			**0.026**
Slow	10 (20.4%)	19 (29.7%)	
Moderate	2 (4.1%)	11 (17.2%)	
Rapid	37 (75.5%)	34 (53.1)	
Post-OP chemo/radiotherapy			**0.002**
No	5 (10.2%)	23 (35.9%)	
Yes	44 (89.8%)	41 (64.1%)	
Other metastasis			0.795
No	31 (63.3%)	42 (65.6%)	
Yes	18 (36.7%)	22 (34.4%)	
Vertebra level			**0.030**
Thoracic	26 (53.1%)	21 (32.8%)	
Lumbar	23 (46.9%)	43 (67.2%)	
Collapse			0.866
No	30 (62.5%)	39 (60.9%)	
Yes	18 (37.5%)	25 (39.1%)	
Posterior wall destruction			**< 0.001**
No	2 (4.1%)	22 (34.4%)	
Yes	47 (95.9%)	42 (65.6%)	
Injected laterality			**0.002**
Single	25 (51.0%)	15 (23.4%)	
Bilateral	24 (49.0%)	49 (76.6%)	
Injected volume	5.75 (4.00–6.00)	4.00 (3.00–6.00)	**0.007**

**Table 3:** Multivariate logistic analysis for the occurrence of cement leakage

Features	OR	(95%CI)	*P*
Tomita classification	2.060	1.124–3.776	**0.019**
Post-OP chemo/radiotherapy	2.679	0.822–8.734	0.102
Vertebra level	0.724	0.232–2.253	0.577
Posterior wall destruction	19.706	3.653–106.297	**0.001**
Injected laterality	0.369	0.118–1.151	0.086
Injected volume	0.698	0.476–1.024	0.066

**Fig. 1:**
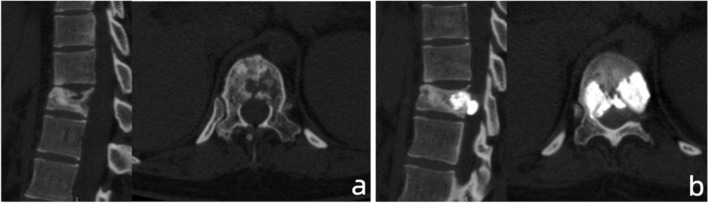
A 27-year-old female patients with breast cancer presented with T11 pathological fracture. **a** Preoperative CT showed the destruction of posterior wall and mild compression of the vertebral body. **b** Postoperative CT presented the leakage of cement into the spinal canal

In a further step, we developed a logistics regression model to investigate the risk factors associated with the leakage into the spinal canal compared to others. As shown in Table 4, posterior wall destruction (*P* = 0.01), injected laterality (*P* = 0.019), and injected volume (*P* = 0.02) could be the potential factors. The multivariate logistic analysis results validated the defects of the posterior wall (OR = 0.121, 95% CI = 0.034-0.423, *P* = 0.001) and injected volume (OR = 1.499, 95% CI = 1.022-2.199, *P* = 0.038) (Table 5) were independently related to the presence of spinal canal leakage (Fig. 1).

**Table 4:** Univariate analysis for the occurrence of cement leakage in the spinal canal

Features	Other = 95	Spinal canal = 18	*P*
Previous chemo/radiotherapy			0.092
No	49 (51.6%)	9 (50.0%)	
Yes	46 (48.4%)	9 (50.0%)	
Tomita classification			0.739
Slow	25 (26.3%)	4 (22.2%)	
Moderate	10 (10.5%)	3 (16.7%)	
Rapid	60 (63.2%)	11 (61.1%)	
Post-OP chemo/radiotherapy			0.359
No	22 (23.2%)	6 (33.3%)	
Yes	73 (76.8%)	12 (66.7%)	
Other metastasis			0.736
No	62 (65.3%)	11 (61.1%)	
Yes	33 (34.7%)	7 (38.9%)	
Vertebra level			0.789
Thoracic	39 (41.1%)	8 (44.4%)	
Lumbar	56 (58.9%)	10 (55.6%)	
Collapse			0.962
No	58 (61.7%)	11 (61.1%)	
Yes	36 (38.3%)	7 (38.9%)	
Posterior wall destruction			**0.001**
No	15 (15.8%)	9 (50.0%)	
Yes	80 (84.2%)	9 (50.0%)	
Injected laterality			**0.019**
Single	38 (40.0%)	2 (11.1%)	
Bilateral	57 (60.0%)	16 (88.9%)	
Injected volume	4.50 (3.00–6.00)	6.00 (4.00–7.12)	**0.020**

**Table 5:** Multivariate logistic analysis for the occurrence of cement leakage in the spinal canal

Features	OR	(95%CI)	*P*
Posterior wall destruction	0.121	0.034–0.423	**0.001**
Injected laterality	3.472	0.642–18.783	0.148
Injected volume	1.499	1.022–2.199	**0.038**

**Fig. 2:**
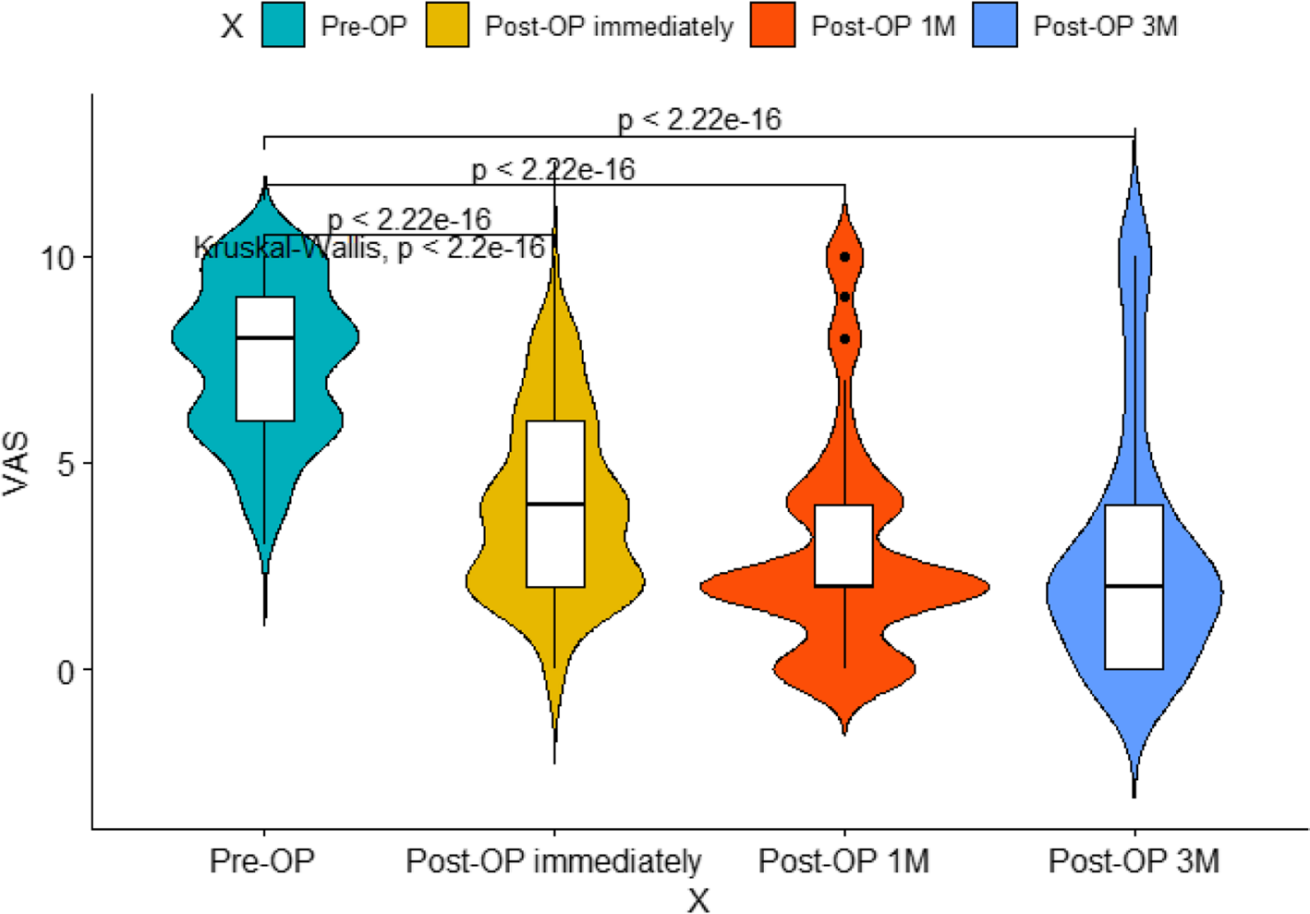
The VAS score before and after surgery

**Fig. 3:**
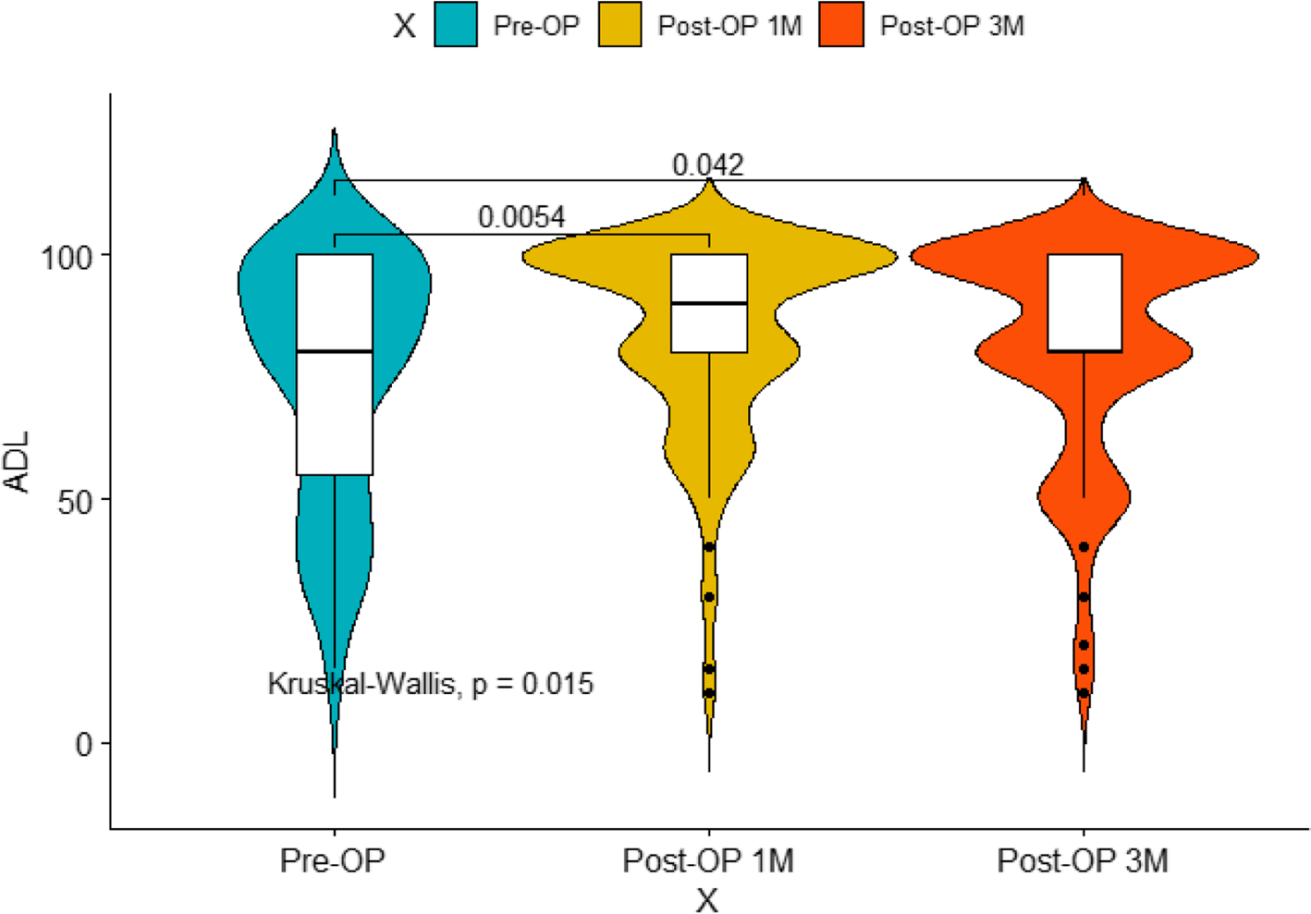
The ADL score before and after surgery

As for the pain management and improvement of ADL, the VAS scores before and after surgery were shown as follows: 8.00 (6.00–9.00) pre-operation, 4.00 (2.00–6.00) immediately after surgery, 2.00 (2.00–4.00) at 1 month after surgery, and 2.00 (0–4.00) at 3 months after surgery. The ADL scores before and after surgery were shown as follows: 80.00 (55.00–100.00) pre-operation, 90.00 (80.00–100.00) at 1 month after surgery, and 80.00 (80.00–100.00) at 3 months after surgery. In detail, 45/64 (70.31%) patients experienced a decrease in VAS scores no less than 3 points after PVP. And as compared to the pre-operative VAS and ADL scores, postoperative evaluation parameters were all showed significant differences (*P* < 0.05) (Figs. 2 and 3). As for the subgroup analysis (Table 6), the preoperative pain scores were 8.00 (6.00–9.75) and 8.00 (6.00–8.00), with no significant difference between them was found (*P* = 0.191). The pain relief after surgery immediately shows no statistical difference (*P* = 0.293): 4.00 (2.00–6.00) and 4.00 (2.00–4.00). Furthermore, the VAS score dropped to 2.00 (2.00–4.00) and 2.00 (2.00–4.00) (*P* = 0.444)1 month following surgery, 2.00 (0.00–4.00) and 2.00 (1.00–3.50) for three months after receiving PVP (Table 6). Apart from this, there was no statistically significant difference in post hoc pain relief scores between 2 sets in each measurement moment. Only a few percent of patients experienced the improvement of daily life: 100.00 (72.50–100.00) and 80.00 (80.00–100.00) (*P* = 0.124) at 1 month postoperatively, 100.00 (70.00–100.00) and 80.00 (80.00–100.00) (*P* = 0.226). Similar to the VAS score, no significant difference was found between the 2 groups.

**Table 6:** The pain management and improvement of ADL

Outcome	Groups		*P*
	Leakage(*n* = 64)	No leakage(*n* = 49)	
Pre-OP VAS score	8.00 (6.00–9.75)	8.00 (6.00–8.00)	0.191
Immediate Post-OP VAS score	4.00 (2.00–6.00)	4.00 (2.00–4.00)	0.293
Post-OP VAS score at 1 month	2.00 (2.00–4.00)	2.00 (2.00–4.00)	0.444
Post-OP VAS score at 3 months	2.00 (0.00–4.00)	2.00 (1.00–3.50)	0.822
Pre-OP ADL score	85.00 (62.50–100.00)	80.00 (52.50–97.50)	0.244
Post-OP ADL score at 1 month	100.00 (72.50–100.00)	80.00 (80.00–100.00)	0.124
Post-OP ADL score at 3 months	100.00 (70.00–100.00)	80.00 (80.00–100.00)	0.226
Post-OP VAS score change after surgery immediately	3.50 (2.00–5.75)	4.00 (2.00–4.00)	0.762
Post-OP VAS score change after 1 month	4.50 (3.00–6.00)	4.00 (3.00–6.00)	0.895
Post-OP VAS score change after 3 months	5.00 (3.00–6.75)	5.00 (2.50–6.00)	0.852
Post-OP ADL score change after 1 month	0.00 (0.00–15.00)	0.00 (0.00–25.00)	0.689
Post-OP ADL score change after 3 months	0.00 (0.00–15.00)	0.00 (0.00–25.00)	0.564

## Discussion

Spine metastases can lead to constant pain at the lesion site, pathological vertebra fractures, and impaired neurological functions [3]. The severe and intractable pain caused by mechanical instability, which was due to loss of spinal integrity as a result of a neoplastic process, can be the main complaints and constraints of daily life such as walking abilities [21]. So, the core of the management of metastatic spinal tumors is to relieve the pain, restore the stability of the spine, prevent the lesion from growing, and improve the quality of life [22]. However, due to the rather low life expectancy and bad personal performance, it is always hard to conduct open surgery for such patients. In contrast, minimally invasive interventions such as radiotherapy, chemotherapy, and PVP/PKP can be more easily acceptable. Compared with nonsurgical procedures, PVP can relieve or eliminate the pain to a large extent, restore the height of vertebrae and slow the progression of the lesion.

According to the reported researches, the main complication of PVP is cement leakage, in which the incidence ranged from 5 to 80%. In the vast majority of clinical studies, cement leakage is asymptomatic and has no clinical consequences. However, some rare and life-threatening complications had been reported. Through the vena cava or paravertebral systems, the leakage could lead to some catastrophic complications such as intracardiac cement embolism, pulmonary embolism, and even death [23–25]. Some other researches also reported that leakage could lead to spinal cord compression or thermal injury [26, 27]. So it is of vital importance to figure out which clinical factors can affect the leakage. Nearly a meta-analysis that enrolled 22 studies validated that intravertebral cleft, cortical disruption, low cement viscosity, and high injected volume of cement could be the high-risk factors for patients with OVCF [16]. However, patients with spine metastases maybe were at increased risk for cement leakage due to umor invasion, bone destruction, and abundant blood supply. On the contrary, very few studies could be found to examine this point.

In our study, cement leakage was found in 64 out of 113 treated vertebrae (56.63%), in which the incidence of each type was shown as below: spinal canal leakage 18 (15.93%), intravascular leakage around the vertebrae 11 (9.73%), and intradiscal and paravertebral leakage 35 (30.97%). Like some prior studies, Zhu SY et al. [28] reported a 64.0% leakage rate for osteolytic metastases, Corcos G et al. [9] found the incidence for osteolytic/mixed spinal metastases was 55%, and Trumm CG et al. [29] showed a 58% leakage rate. Whereas, we also found that the incidence of our study is much higher than other researches. Zhang TY et al. [30] reported a 32.5% and Cui Y et al. [31] reported a 34.9% leakage rate. These findings could be underestimated due to the lack of CT examination after surgery.

Of all the candidate metrics in our study, defects of the posterior wall and Tomita classification were validated as the independent risk factors of leakage in general, as reported previously by Cui Y et al. [28, 31]. Then, we conducted a further study to identify the risk factors of spinal canal leakage due to the other two subtypes were usually asymptomatic. The subtype of spinal canal leakage can result in the compression of the spinal cord or nerve roots. Multivariate logistic analysis showed injected volume of cement, and the incompleteness of the posterior wall was validated as the independent factors for the high incidence of leakage. Although a 15.93% leakage rate was reported in our study, no neurological deficits were found in the treated patients.

According to prior studies, the first and most common site of involvement is the posterior half of the vertebral body, which contributes to the destruction of posterior wall [32]. So previous researches suggested that PVP should be taken seriously for vertebrae with posterior wall defects. In a study conducted by Sun HP et al. [15], it is of high risk to perform PVP for vertebra metastases with destructions of posterior wall, increasing with the degree of posterior wall defects. However, the presence of posterior wall defects did not impact the prognosis. Different from Corcos G et al. [9] had reported, they found that cement leakage could be avoided or controlled by taking care when the cement reaches the posterior wall. Some surgical skills had been reported to reduce the possibility of leakage. Sun HP et al. [15] suggested that orthopedic surgeons could inject a little cement in the perilesional normal areas to prevent the displacement of cement. Yang HL et al. introduced another surgical procedure in which the cement injection should stop in time when it reaches the lateral margin or when one-fourth of the distance to the posterior wall of vertebra left [33]. Another challenging surgical method suggested by them is blocking the defect areas with high-viscosity cement [33]. It was also reported that the morphology of the posterior wall in thoracic vertebrae may had potential association with the incidence of spinal canal leakage, and the posterior 1/6 of the vertebral body should be taken as the finish line to avoid further cement displacement [24].

According to Tomita classification, the primary tumors were categorized as three subtypes based on the speed of growth [18]. However, there are little researches to further investigate the association between the type of tumor and leakage rate. Corcos G et al. [9] reported a lower vascular cement leakage rate in lung cancer patients and attributed this phenomenon to the intravertebral pressure which was relied on the density of cells and the type of histologies. Some other researchers had also found out that metastasis of breast cancer is more likely to encounter the leakage of cement [31]. The leakage through the intravascular approach could be divided into several ways: (1) segmental vertebral veins; (2) anterior external vertebral venous plexus; (3) tumor reflux vein. According to previous researches [34, 35], the destruction of the venous system due to vertebral fractures could be the protective factor of cement leakage. Moreover, the destruction of the posterior vertebral wall, which resulted from tumor invasion, was widely considered a risk factor of cement leakage. In the vast majority of cases, the severity of tumors’ invasion was closely related to the abundant blood supply and the tumor reflux vein [31]. We suppose that the growth speed of the tumor can reflect the severity and blood supply of the tumor, which may contribute to the high rate of cement leakage. Unfortunately, no digital subtraction angiography (DSA) was performed for patients. Besides that, we could not analyze the effect of each exact tumor type on leakage due to the small-sample study. So, more clinical experiments are needed to investigate the potential links.

Highly associated with the maintenance and restoration of vertebrae height, the cement volume of injection was considered to be associated with the leakage in many previous studies [9, 16, 25, 31, 36]. Surprisingly, no association was found in many studies between the injected volume and pain management after PVP [37, 38]. So a large amount of cement injection will not contribute to the benefit of surgery; instead, it may increase complications. According to the experience reported by Zhu SY et al. [28], less than 3.5 ml of cement in thoracic vertebrae and 4ml in lumbar vertebrae are safe to prevent leakage. Similar to the finding by Chew, equal to or greater than 4 ml of cement volume may lead to a higher complication rate [39]. To balance the pain management and leakage, the study conducted by Nieuwenhuijse et al. reported that intravertebral cement volume of an optimal value of 24% is a safe threshold [40]. Therefore, surgeons should balance the benefits and harms carefully during the whole procedure.

Recently, a meta-analysis confirmed that PVP could effectively relieve the pain in patients with spine metastases, based on the data of 22 included researches [13]. According to one previous study, an average of 3.2 score improvement of VAS score was reported [41]. Our result coincided with some prior studies [42]. Surprisingly, no significant differences were found in every follow-up time between the leakage group and the non-leakage group. Nevertheless, we can conclude that PVP can be an effective way to manage pain based on the data. Moreover, pain relief can be achieved immediately after the surgery and last a long time. Unlike pain management, the improvement of ADL seemed to be less. We ascribe this to several reasons. Firstly, multiple other bone metastases outside the surgical site may still limit the activities of daily life. Second, various comorbidities would be the restriction of improvement of outcomes.

In some recent studies, patients with a lower filling rate (less than 0.646) of vertebrae and injected volume of cement were more likely to suffer from local bone destruction progression (LBDP) within 6 months [43], which would be a divergence from the original intent of the PVP procedure. However, in our analysis, the injected volume of cement was strongly associated with the occurrence of cement leakage. Previous literature on the subject has also suggested the same. Nevertheless, the cement killed tumor cells by the heat generated in the process of polymerization, and the higher amount of cement injected, the higher temperature reached, according to the principle [44]. In other words, the amount of cement is positively correlated with the efficiency of local tumor control and the interval to LBDP. What is apparent is that as the amount of bone cement used increases, the incidence of cement leakage and other complications also increases. To address this issue, some novel combined surgery had been introduced to reduce tumor volume and control the injected volume of cement safely, such as radiofrequency ablation (RFA) and ^125^I seed implantation [10–12]. However, these technologies are still at the initial and exploratory stage in the current situation. Further research will be needed to allow for greater generalization and application. So, a better control of cancer progression and a lower risk of cement leakage were conflicting and controversial. In clinical practice, the decision was largely dependent on the clinical experience and awareness of the surgeon, which resulted in a lack of consensus on this point. Therefore, in the future, a large-sample multicenter study with a long-term follow-up is required to determine an optimal injected volume range for patients with spine metastases.

Despite that, our research had several limitations. First and foremost, the retrospective nature of the small-sample study may be associated with bias. Secondly, there are still some factors that were not included in this study, such as the viscosity of cement, the time of surgery, and so on. Thirdly, only thoracic and lumbar spines were included in our study.

## Conclusion

The leakage of cement is the most frequent and common complication for metastatic spinal tumors after PVP. In our study, Tomita classification and the destruction of the posterior wall were independent risk factors for leakage in general. And defects of the posterior wall and injected volume were independently related to the presence of spinal canal leakage. The PVP procedure can be an effective way to manage the pain.

## Data Availability

The datasets generated and/or analyzed during the current study are not publicly available due to the data is confidential patient data but are available from the corresponding author on reasonable request.

## References

[1] Barzilai O, Laufer I, Yamada Y, Higginson DS, Schmitt AM, Lis E, Bilsky MH (2017). Integrating evidence-based medicine for treatment of spinal metastases into a decision framework: neurologic, oncologic, mechanicals stability, and systemic disease. J Clin Oncol.

[2] Kassamali RH, Ganeshan A, Hoey ETD, Crowe PM, Douis H, Henderson J (2011). Pain management in spinal metastases: the role of percutaneous vertebral augmentation. Ann Oncol.

[3] Chiu RG, Mehta AI (2020). Spinal Metastases. JAMA.

[4] Spratt DE, Beeler WH, de Moraes FY, Rhines LD, Gemmete JJ, Chaudhary N, Shultz DB, Smith SR, Berlin A, Dahele M (2017). An integrated multidisciplinary algorithm for the management of spinal metastases: an International Spine Oncology Consortium report. Lancet Oncol.

[5] Yang Z, Yang Y, Zhang Y, Zhang Z, Chen Y, Shen Y, Han L, Xu D, Sun H (2015). Minimal access versus open spinal surgery in treating painful spine metastasis: a systematic review. World J Surg Oncol.

[6] Muijs SP, Nieuwenhuijse MJ, Van Erkel AR, Dijkstra PD (2009). Percutaneous vertebroplasty for the treatment of osteoporotic vertebral compression fractures: evaluation after 36 months. J Bone Joint Surg (Br).

[7] Galibert P, Deramond H, Rosat P, Le Gars D (1987). Preliminary note on the treatment of vertebral angioma by percutaneous acrylic vertebroplasty. Neuro-Chirurgie.

[8] Xin X, Feng J, Yue C, Jin T, Liu X (2019). Monostotic fibrous dysplasia at C7 treated with vertebroplasty: a case report and review of the literature. World J Surg Oncol.

[9] Corcos G, Dbjay J, Mastier C, Leon S, Auperin A, De Baere T, Deschamps F (2014). Cement leakage in percutaneous vertebroplasty for spinal metastases: a retrospective evaluation of incidence and risk factors. Spine.

[10] Xie L, Chen Y, Zhang Y, Yang Z, Zhang Z, Shen L, Yuan Z, Ren M (2015). Status and prospects of percutaneous vertebroplasty combined with ^125^I seed implantation for the treatment of spinal metastases. World J Surg Oncol.

[11] Yang Z, Zhang Y, Xu D, Maccauro G, Rossi B, Jiang H, Wang J, Sun H, Xu L, Chen Y (2013). Percutaneous vertebroplasty combined with interstitial implantation of 125I seeds in banna mini-pigs. World J Surg Oncol.

[12] Katonis P, Pasku D, Alpantaki K, Bano A, Tzanakakis G, Karantanas A (2009). Treatment of pathologic spinal fractures with combined radiofrequency ablation and balloon kyphoplasty. World J Surg Oncol.

[13] Qi L, Li C, Wang N, Lian H, Lian M, He B, Bao G (2018). Efficacy of percutaneous vertebroplasty treatment of spinal tumors: A meta-analysis. Medicine.

[14] Schmidt R, Cakir B, Mattes T, Wegener M, Puhl W, Richter M (2005). Cement leakage during vertebroplasty: an underestimated problem?. Eur Spine J.

[15] Sun H, Yang Z, Xu Y, Liu X, Zhang Y, Chen Y, Xu D, Yang Y, Li D, Xia J (2015). Safety of percutaneous vertebroplasty for the treatment of metastatic spinal tumors in patients with posterior wall defects. Eur Spine J.

[16] Zhan Y, Jiang J, Liao H, Tan H, Yang K (2017). Risk factors for cement leakage after vertebroplasty or kyphoplasty: a meta-analysis of published evidence. World Neurosurg.

[17] Leithner A, Radl R, Gruber G, Hochegger M, Leithner K, Welkerling H, Rehak P, Windhager R (2008). Predictive value of seven preoperative prognostic scoring systems for spinal metastases. Eur Spine J.

[18] Tomita K, Kawahara N, Kobayashi T, Yoshida A, Murakami H, Akamaru T (2001). Surgical strategy for spinal metastases. Spine.

[19] Hamill-Ruth RJ, Marohn ML (1999). Evaluation of pain in the critically ill patient. Crit Care Clin.

[20] Rollnik JD (2009). Barthel index as a length of stay predictor in neurological rehabilitation. Die Rehabil.

[21] Fisher CG, DiPaola CP, Ryken TC, Bilsky MH, Shaffrey CI, Berven SH, Harrop JS, Fehlings MG, Boriani S, Chou D (2010). A novel classification system for spinal instability in neoplastic disease: an evidence-based approach and expert consensus from the Spine Oncology Study Group. Spine.

[22] Lin M, Qu M, Huang W, Liu T, Duan R, Yuan Y, Gao J, Zhang M, Yu X (2021). Clinical effectiveness of percutaneous vertebroplasty in conjunction with postoperative radiotherapy in the treatment of spinal metastases. J Cancer Res Clin Oncol.

[23] Fadili Hassani S, Cormier E, Shotar E, Drir M, Spano JP, Morardet L, Collet JP, Chiras J, Clarençon F (2019). Intracardiac cement embolism during percutaneous vertebroplasty: incidence, risk factors and clinical management. Eur Radiol.

[24] Zhang S, Wang GJ, Wang Q, Yang J, Xu S, Yang CH (2019). A mysterious risk factor for bone cement leakage into the spinal canal through the Batson vein during percutaneous kyphoplasty: a case control study. BMC Musculoskelet Disord.

[25] Hsieh MK, Kao FC, Chiu PY, Chen LH, Yu CW, Niu CC, Lai PL, Tsai TT. Risk factors of neurological deficit and pulmonary cement embolism after percutaneous vertebroplasty. J Orthop Surg Res. 2019;14(1):406. 10.1186/s13018-019-1459-4 PMC6884871 31783861

[26] Lai PL, Tai CL, Chen LH, Nien NY (2011). Cement leakage causes potential thermal injury in vertebroplasty. BMC Musculoskelet Disord.

[27] Teng MM, Cheng H, Ho DM, Chang CY (2006). Intraspinal leakage of bone cement after vertebroplasty: a report of 3 cases. AJNR Am J Neuroradiol.

[28] Zhu S-Y, Zhong Z-M, Wu Q, Chen J-T (2016). Risk factors for bone cement leakage in percutaneous vertebroplasty: a retrospective study of four hundred and eighty five patients. Int Orthop.

[29] Trumm CG, Pahl A, Helmberger TK, Jakobs TF, Zech CJ, Stahl R, Paprottka PM, Sandner TA, Reiser MF, Hoffmann RT (2012). CT fluoroscopy-guided percutaneous vertebroplasty in spinal malignancy: technical results, PMMA leakages, and complications in 202 patients. Skelet Radiol.

[30] Zhang TY, Zhang PX, Xue F, Zhang DY, Jiang BG. Risk factors for cement leakage and nomogram for predicting the intradiscal cement leakage after the vertebra augmented surgery. BMC Musculoskelet Disord. 2020;21(1):792. 10.1186/s12891-020-03810-4 PMC7702672 33256689

[31] Cui Y, Pan Y, Lin Y, Mi C, Wang B, Shi X. Risk factors for predicting cement leakage in percutaneous vertebroplasty for spinal metastases. J Orthop Sci. 2022;27(1):79–83. 10.1016/j.jos.2020.10.004 33158733

[32] Algra PR, Heimans JJ, Valk J, Nauta JJ, Lachniet M, Van Kooten B (1992). Do metastases in vertebrae begin in the body or the pedicles? Imaging study in 45 patients. AJR Am J Roentgenol.

[33] Yang H, Pan J, Sun Z, Mei X, Yang Y (2012). Percutaneous augmented instrumentation of unstable thoracolumbar burst fractures: our experience in preventing cement leakage. Eur Spine J.

[34] Ding J, Zhang Q, Zhu J, Tao W, Wu Q, Chen L, Shi P, Zhang H (2016). Risk factors for predicting cement leakage following percutaneous vertebroplasty for osteoporotic vertebral compression fractures. Eur Spine J.

[35] Nieuwenhuijse MJ, Van Erkel AR, Dijkstra PD (2011). Cement leakage in percutaneous vertebroplasty for osteoporotic vertebral compression fractures: identification of risk factors. Spine J.

[36] Shi X, Cui Y, Pan Y, Wang B, Lei M (2021). Epidemiology and detection of cement leakage in patients with spine metastases treated with percutaneous vertebroplasty: a 10-year observational study. J Bone Oncol.

[37] Kaufmann TJ, Trout AT, Kallmes DF (2006). The effects of cement volume on clinical outcomes of percutaneous vertebroplasty. AJNR Am J Neuroradiol.

[38] Knavel EM, Rad AE, Thielen KR, Kallmes DF (2009). Clinical outcomes with hemivertebral filling during percutaneous vertebroplasty. AJNR Am J Neuroradiol.

[39] Chew C, Craig L, Edwards R, Moss J, O'Dwyer PJ (2011). Safety and efficacy of percutaneous vertebroplasty in malignancy: a systematic review. Clin Radiol.

[40] Nieuwenhuijse MJ, Bollen L, van Erkel AR, Dijkstra PD (2012). Optimal intravertebral cement volume in percutaneous vertebroplasty for painful osteoporotic vertebral compression fractures. Spine.

[41] Bae JW, Gwak HS, Kim S, Joo J, Shin SH, Yoo H, Lee SH (2016). Percutaneous vertebroplasty for patients with metastatic compression fractures of the thoracolumbar spine: clinical and radiological factors affecting functional outcomes. Spine J.

[42] Fourney DR, Schomer DF, Nader R, Chlan-Fourney J, Suki D, Ahrar K, Rhines LD, Gokaslan ZL (2003). Percutaneous vertebroplasty and kyphoplasty for painful vertebral body fractures in cancer patients. J Neurosurg.

[43] Liu Z, Liang H, Sun W, Lu Z, Pan S (2021). Risk factors for local bone destruction progression in palliative percutaneous vertebroplasty for vertebral metastases and the significance of bone cement filling rates. Pain Physician.

[44] Stańczyk M, van Rietbergen B (2004). Thermal analysis of bone cement polymerisation at the cement-bone interface. J Biomech.

